# OpenVNT: An Open Platform for VIS-NIR Technology

**DOI:** 10.3390/s23063151

**Published:** 2023-03-15

**Authors:** Roman-David Kulko, Alexander Pletl, Heike Mempel, Florian Wahl, Benedikt Elser

**Affiliations:** 1 Technologie Campus Grafenau, Technische Hochschule Deggendorf, 94481 Grafenau, Germany; 2 Institut für Gartenbau, Hochschule Weihenstephan-Triesdorf, 85354 Freising, Germany

**Keywords:** visible and near infrared spectroscopy, open source, instrumentation, spectrometer, chemometrics, nondestructive evaluation, fruit quality

## Abstract

Spectrometers measure diffuse reflectance and create a “molecular fingerprint” of the material under investigation. Ruggedized, small scale devices for “in-field” use cases exist. Such devices might for example be used by companies in the food supply chain for inward inspection of goods. However, their application for the industrial Internet of Things workflows or scientific research is limited due to their proprietary nature. We propose an open platform for visible and near-infrared technology (OpenVNT), an open platform for capturing, transmitting, and analysing spectral measurements. It is built for use in the field, as it is battery-powered and transmits data wireless. To achieve high accuracy, the OpenVNT instrument contains two spectrometers covering a wavelength range of 400–1700 nm. We conducted a study on white grapes to compare the performance of the OpenVNT instrument against the Felix Instruments F750, an established commercial instrument. Using a refractometer as ground truth, we built and validated models to estimate the Brix value. As a quality measure, we used coefficient of determination of the cross-validation ($R^{2}CV$) between the instrument estimation and ground truth. With 0.94 for the OpenVNT and 0.97 for the F750, a comparable $R^{2}CV$ was achieved for both instruments. OpenVNT matches the performance of commercially available instruments at one tenth of the price. We provide an open bill of materials, building instructions, firmware, and analysis software to enable research and industrial IOT solutions without the limitations of walled garden platforms.

## 1. Introduction

Spectroscopy in the visible and near-infrared (VIS-NIR) range detects the absorption spectra of the material under investigation. As each molecule absorbs a certain wavelength range in a unique way, the technique is said to yield a “molecular fingerprint”. In the case of simple and less complex samples, this ’molecular fingerprint’ can also be used directly. However, the technique is also applied to highly complex samples such as fruits. In such cases, special methods are required for the analysis of the spectral data. In combination with machine learning methods, it has been used for decades to assess the quality and determine the ripeness of fruit and vegetables [1]. For this purpose, special handset spectrometers have been developed in addition to larger laboratory equipment. The former are often referred to as near-infrared (NIR) scanners. Due to their design, NIR scanners for in-field use need to be simplified in comparison to larger laboratory instruments and thus are tailored towards specific applications. For example, to analyse fruit and vegetables, low-cost silicon sensors are usually integrated. Depending on the configuration of the instrument, these sensors detect from visible (VIS) to near-infrared (NIR) electromagnetic radiation (400–1100 nm).

The availability of those inexpensive, small-scale devices has led to scientific interest in different areas. Part of that research focuses on the quality of the produced fingerprint [2] and compares the different devices. Another part strives to apply the advances of machine learning and artificial intelligence techniques to improve the quality of low-cost instruments towards that of their more expensive counterparts [3]. Finally, there is research that focuses on the integration of the technology into specific workflows. Access to raw data is the basis for such research, but data has increasingly become a value on its own. At present, manufacturers are pushing proprietary cloud platforms that are beginner-friendly but only allow data to be exported to a limited extent. These walled gardens hold back scientific research as well as tight integration into industrial internet of things (IIOT) use cases.

Therefore, we present OpenVNT comprising an OpenVNT instrument, a sample dataset, as well as a toolbox for building machine learning models for material analysis. OpenVNT achieves a performance comparable to commercial in-field NIR scanners, while preserving complete data sovereignty by open sourcing hard and software components.

We originally built the platform for an IIOT use-case within a company operating in the food supply chain. Assessing the quality measures of a product in an automated fashion is key for data-driven business use-cases, such as the creation of digital twins, or shelf life prediction. Ideally, data collection should occur upon the receipt of goods. However, companies in the food supply chain collect few samples because of the time-consuming nature of measurements using conventional laboratory equipment, such as refractometers. Such data are frequently recorded using pen and paper. For those companies, the process is slow, error-prone and does not provide a sufficient volume of data-driven decisions.

The availability of OpenVNT as an open platform and the integration in the companies workflow produces the same quality information as with refractometers, while producing more data in the aforementioned use-case.

In order to make use of the huge benefit of these systems, proper modelling and careful maintenance is necessary. Model performance depends on data quality and consistency. Both can change over time, e.g., due to changes in the instrument or in the environment. However, seasonal variations in samples can also cause the model to need to be recalibrated.

Research on NIR scanners falls into roughly three categories: The analysis and assessment of NIR scanner hardware, application of scanners and their performance in specific applications and the introduction of do-it-yourself devices. Independent of the research focus, the procedure for taking VIS-NIR measurements, model building and validation are common among them. We will introduce them as follows.

There are several instruments on the market. Zhu et al. [4] reviews the main types of commercial universal serial bus (USB) NIR spectrometers and compares their specifications. These devices are used in order to perform nondestructive quality assessment, e.g., food and especially fruits. Xie et al. [5] reviews the applications of NIR systems for the quality evaluation of fruits.

USB spectrometers are designed to work in a laboratory environment and require additional peripherals, e.g., light source, probe head, etc.. They are typically driven by a PC using proprietary software. Such a setup is used, e.g., by Xiao et al. [6] for the quality assessment and discrimination of intact white and red grapes.

Some of the USB spectrometers could be adapted to portable field applications. For example, Sun et al. [7] demonstrated the use of an USB spectrometer as a portable device for detecting fruit quality by diffuse reflectance VIS-NIR spectroscopy. Yang et al. [8] reported using an USB spectrometer as a portable and nondestructive detector for estimating sugar content in kiwifruits.

Specialised commercial devices, so-called NIR scanners, are handheld battery-powered spectrometers. They contain a light source, are designed to work in-field applications, and can store measurements on the device. Three NIR scanners are commonly mentioned: the F750 by Felix Instruments [9], the H100 by Sunforest [10] and the Scio by Consumer Physics [11]. Goisser et al. [12] investigated the use of all three NIR scanners and their applications in the fruit and vegetable sector. Kaur et al. [13] compared NIR scanners for the assessment of fruit dry matter. Pu et al. [14] reviewed recent advances in portable and handheld NIR spectrometers and applications in milk, cheese, and dairy powders. Marques et al. [15] investigated the performance of handheld NIR spectrometers for the nondestructive analysis of umbu fruit quality. Other companies that offer NIR scanners are: StellarNet Inc. [16], Solid Scanner [17], and GoyaLab [18].

Finally, there are self-made handheld instruments. To the best of our knowledge, there is only one self-built battery driven handheld instrument described in the literature. Guo et al. [19] reported a novel noninvasive and cost-effective handheld detector to determine soluble solids content of fruits. They integrated a commercial-grade USB spectrometer with a wavelength range of 650–1100 nm into their handheld instrument as well as a light source, battery, and single-board computer.

The operating principle of some NIR scanners, which like OpenVNT, are based on spectrometers using a grating monochromator, consists of five steps: (1) A light source, usually a light bulb or led, irradiates the sample, causing characteristic diffuse reflections to occur within the sample; (2) Reflected radiation is guided into the spectrometer via light guides, optical fibre; (3) In the spectrometer, reflected radiation is split into wavelength ranges by means of a grating and projected onto an light sensitive electronic sensor; (4) The electronic sensor generates a signal proportional to the radiation intensity per pixel, an intensity spectrum; (5) Finally, micro-controllers are used to generate spectral data and digital signal processing computes the desired output. Resulting spectral data are subsequently fed as input for a chemometric model, which estimates the targeted parameter, e.g., the Brix value, to quantify the soluble solid content (ssc) of the sample.

Measurements with classic laboratory equipment, e.g., refractometers, are considered ground truth and serve as reference values for model building. However, such methods are slower and destructive for the object under investigation and are unsuitable for a production environment.

Resulting regression models aim to predict reference values from spectral data. The model quality is assessed as the deviation between the estimated and reference values [20,21]. Typically, a residual-based metric is used to gauge the model performance, as can be seen in Section 2.8.

Estimation models can be built using statistical/machine learning techniques. Historically, statistical methods such as principal component analysis regression were used in addition to simple and multiple linear regression models.

At present, partial least squares regression (PLSR) is considered the standard regression method [22,23]. Parameterising PLSR models requires data preprocessing to minimise physical effects, e.g., scattering, etc., on spectral data that can hamper correlation. There are a variety of corrections and their combinations. Hence, preprocessing requires domain-specific knowledge. Rinnan et al. [24] reviews most of common preprocessing techniques for near-infrared spectra. One common preprocessing method for VIS-NIR spectral data is the Savitzky–Golay filter [25]. Cui et al. [26] reports an investigation of the use of convolutional neural networks (CNN) for multivariate regression on NIR data. They also compare the performance between the CNN method and PLSR method using different data sets. In recent years, these novel deep learning algorithms have gained popularity. Ng et al. [27] used one-dimensional CNN for the simultaneous prediction of several soil properties VIS-NIR and mid-infrared spectra. Kawamura et al. [28] used a one-dimensional CNN on VIS-NIR spectroscopy to improve the soil phosphorus prediction.

The advantage of CNN over established procedures is that virtually no prior knowledge of physical effects and preprocessing is necessary. Due to the independent learning of the deep learning approach, regressions and classifications are generated that are generally not significantly better than those with sophisticated preprocessing.

One of commonly used classification algorithms is linear discriminant analysis (LDA) [1,29]. Cortés et al. [30,31] usedLDA, among other classification models, to discriminate sweet and nonsweet nectarines using visible and near-infrared spectroscopy. Acqua et al. [32] used the NIR spectroscopy and LDA for the discrimination intact fruit of euterpe oleracea and edulis.

All three mentioned types of devices suffer one main disadvantage: a lack of automation. For creating business value, measurement data have to be fed into a downstream processes. A manual exchange of data between the spectrometers software and target process interrupts the work flow. To overcome this major disadvantage, one has to invest in additional software or hardware development. Therefore, we decided to develop a relatively simple device from the ground up, with a focus on maximal data sovereignty and communication flexibility. Our solution allows fast data communication into systems such as enterprise resource planning systems. As such, traditional pen and paper processes can be replaced by a more efficient digital counterpart. Furthermore, the availability of data lays the foundation for advanced usages, such as data-driven decision making, demand forecasting algorithms based on quality information or dynamic pricing algorithms [33,34].

The remainder of the work is structured as follows: First, we introduce the related work in Section 1. Section 2 describes the hardware design of the OpenVNT instrument and the architecture of the software. Finally, the chemometric models drawn from our proprietary instrument are presented in Section 2.5 to assess the quality of the OpenVNT. We discuss our results in Section 4 and give an outlook on future work in Section 5.

## 2. Materials and Methods

We use spectral data recorded by the F750 as benchmark in Section 2.5. The F750 was sourced at Felix Instruments, Vancouver, WA, United States of America [9]. According to Felix Instruments, the F750 uses a Carl Zeiss MMS-1 NIR enhanced spectrometer. The MM1 spectrometer covers a wavelength range from 310 to 1100 nm with 10 nm spectral resolution full width at half maximum (FWHM), 256 pixel, 3.3 nm/pixel and a signal-to-noise ratio (SNR) of 11,600 [35]. A xenon tungsten light bulb was used as a light source. The price of the F750 including the software and model builder was approximately about EUR 8000.

### 2.1. Requirements

Our goal for the OpenVNT platform was to offer a solution for field use that covers a wide range of applications and provides collected measurements to the user in an automated fashion. Thus, we identified the following hardware, sensing, and software requirements:

#### 2.1.1. Hardware Requirements

First of all, our device should be built for use in the field, outside of the lab. Thus, we aim at a portable, lightweight, handheld device. The OpenVNT instrument should be operated using one hand, have a display for status indication. For recharging and software updates, a USB Type-C (USB-C) connector should be used.

To enable data collection in the field, three basic requirements need to be met in order to allow for truly wireless operation: (1) The device has to be battery powered, such that at least 200 measurements can be taken with a charged battery. (2) Measurement data have to be communicated through wireless communication channels, e.g., bluetooth low energy (BLE) and/or wireless local area network (WLAN). (3) At first, a single measurement, including data handling and obtaining a result, has to be taken in under 10 s, but this should be improved to approximately 2 s.

#### 2.1.2. Sensing Requirements

In order to cover a wide range of applications, we aim for a wide range of wavelengths covered by the spectrometer(s), in our case, a range of 350 nm to 1700 nm. In addition, a SNR greater than 5000:1 and a FWHM of less than 16 nm spectral resolution is required. These requirements reflect typical specifications of original equipment manufacturer (OEM).

#### 2.1.3. Software Requirements

In order to attract a large audience of potential programmers, we decided to use MicroPython as the programming language of choice. Since Python is already popular in the scientific computing and data analytics communities, we found it to be a good fit. Furthermore, measurement methods and communication protocols must be customisable for users of the OpenVNT platform. As default, measurements should be communicated in the popular java script object notation (JSON) format.

### 2.2. Hardware

The OpenVNT instrument consists of commercially available components, listed in Table 1. Approximate prices for each component are also listed. In total, the material costs were approximately EUR 3400. Depending on the application of the OpenVNT, the cost of driving the InGaAs-based Nir22 spectrometer can be left out. In this case, using only a Si-based Vis29 spectrometer, material costs are decreased to approximately EUR 900.

The OpenVNT instrument includes two spectrometers. Its VIS spectrometer covers a wavelength range from 148 nm to 1161 nm with a spectral resolution of 10 nm FWHM, at 512 pixels with 2 nm/pixel and a SNR >5000. Its NIR spectrometer covers a wavelength range from 877 nm to 1902 nm with a spectral resolution of <16 nm FWHM, at 128 pixels at 8.2 nm/pixel and a SNR >5000. Both spectrometers were sourced from Dr. Licht GmbH [36]. Furthermore, the NIR spectrometer offers regulated lamp control outputs, which enable voltage regulation and switching the light source on and off. A xenon tungsten light bulb, integrated into a reflector to focus the radiation, is used as the light source. A M5Stack Fire based on an ESP32 micro-controller serves as the processing and communication unit. Communication between the micro-controller and the spectrometers is performed using universal asynchronous receiver-transmitter (UART). To communicate with controlling devices, e.g., smartphones or networked PCs, the micro-controller offers connections via BLE or WLAN. Two lithium-ion batteries are connected in parallel to provide power. A boost converter is used for charging the battery and converting the battery voltage to a stable 5.2 V. For charging and firmware upload, a USB-C socket is used. Lastly, a switch is included to power off the OpenVNT instrument. Housing parts, lamp holders, and M5Stack holders are 3D-printed using polylactic acid (PLA) filament. Open source Firmware, schematics, 3D CAD models, and a tutorial on how to build the OpenVNT instrument are available online (https://mygit.th-deg.de/vnt, accessed on 10 March 2023).

**Figure 1:**
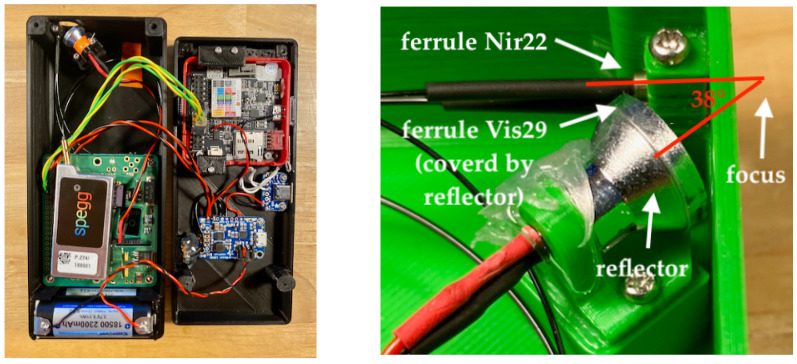
The OpenVNT hardware. **Left**: Inside view of the instrument. **Right**: Probe head.

Part of the OpenVNT instrument hardware is shown in Figure 1. The left image shows the inside of the instrument. Visible are the top of the stacked two spectrometers, a silver box with label “spegg”, batteries, larger blue printed circuit board (pcb) is the boost converter, smaller blue pcb is the USB-C Breakout Board, and M5Stack Fire with a red plastic frame and the probe head.

The right image of Figure 1 shows an image of the probe head in detail. The probe head is of critical importance. It is designed to measure diffuse reflectance in interactance mode [37]. The lamp and the metal housed ends of the optical fibre and ferrules are combined in the probe head. The ferrule of the Nir22 is in the same plane as the lamp. The ferrule of the Vis29 is offset 2 mm downwards. Both ferrules end in the same plane and have a diameter of 300 µm. The angle between the lamp axis and the Nir22 fibre is roughly 38°. This angle resulted under the circumstances of the focus point of the reflector and the housing. The focal point of the lamp is 5 mm in front of the fibre ends and 15 mm away from the lamp. This geometry allows the ideal illumination of the sample in contact with the probe head. The arrangement was designed so that the two ends of the ferrules can be slightly pressed into a soft sample by 0.25 mm. This ensures that measurements are made exclusively in interactance mode.

### 2.3. Software

The software is organised in layers: (1) Communication; (2) Hardware abstraction; (3) User interface; and (4) Controller. A configuration file is used to select the spectrometers and some functions of the OpenVNT. This defines a variety of parameters, such as the number of averages of the spectral data.

#### 2.3.1. Communication

The communication module connects the ESP32 to a smart device (phone, tablet) controlling the spectrometer. Control of the smart device can be integrated into the downstream process using domain specific applications, such as an “app” for managing a warehouse. This is based on a collection of standard MicroPython functions and a BLE class. To save as much power as possible, we used bluetooth low energy. As an alternative communication method, WLAN and message queueing telemetry transport (MQTT) can also be used (not further described).

#### 2.3.2. Hardware Abstraction

The hardware abstraction layer contains classes for controlling hardware components. These include the two spectrometers Vis29 and Nir22 as well as optional components, e.g., external lamps or temperature sensors. The spectrometer classes include methods for communication between ESP32 and the spectrometer via UART. Measurement methods for the acquisition of spectral data and their transformations are also implemented. The hardware layer includes a mock-spectrometer which returns predefined values from the configuration source for testing purposes.

#### 2.3.3. User Interface

The user interface module content allows the user to interact with the instrument. It contains classes for controlling physical buttons and the display of the M5Stack Fire. Since the state of the instrument is controlled by a coupled smart BLE device, the user interface only supports a limited set of actions. This displays the current step in the measurement procedure and activates or deactivates buttons. Typical interaction on the device are initiating measurements, starting the calibration or displaying spectra of the last measurement. If user interaction without a BLE device is desired, we provide an implementation necessary to control the states, buttons and display of the M5Stack Fire.

#### 2.3.4. Controller

The controller module basically links all modules together. In doing so, it performs the following tasks: (1) Managing device states; (2) Responding to user commands; (3) Executing measurement procedures (calibration, sample measurement); (4) Sending (and saving) measurement results and device information.

### 2.4. Measurement Procedure

The spectrometer class contains methods for acquiring spectral data and setting spectrometer parameters such as the integration time, averaging and gain. In order to obtain a measurement from a sample, four process steps are necessary for a measurement:

(1) A measurement is performed at an empirically set integration time; (2) The recorded intensity spectrum is checked for saturation of the sensor; (3) The optimal integration time is determined and set as the new integration time; (4) Finally, the actual measurement is performed.

Temperature-dependent drift of the spectrometers is not compensated by hardware or software. However, the drift of the Vis29 is generally negligible. In the case of the Nir22, we accept a minimal drift.

#### 2.4.1. Integration Time Optimiser

Classically, a measurement is performed on a sample using the spectrometer settings that are set for the reference, e.g., Teflon. Using this method, the intensity of the spectral data may be very small because the diffuse reflectance from a sample can be much lower than that from the reference. To ensure a high intensity of the spectral data and at the same time an optimal SNR for each measurement, it is advisable to adjust the integration time (and to increase the number of averages). Finding the optimal integration time, $it_{opt}$, is achieved as follows:

(1) An intensity spectrum is measured at a typical integration time; (2) The reflectance is calculated; (3) The maximum value of reflectance is determined; (4) $it_{opt}$ is obtained by dividing typical integration time by maximum of reflectance; (5) Optional: Correct $it_{opt}$ by multiplying $it_{opt}$ by an empiric factor of 0.90 to ensure reflectance below 1; (6) Check if $it_{opt}$ is below a threshold of 5 s, in order to avoid large measurement times or sensor saturation; and 7) Finally, repeat measurement with $it_{opt}$.

#### 2.4.2. Calibration

The calibration measurement is necessary as the instrument needs information as spectral references.

These reference spectra were recorded using semi-transparent Teflon block as sample (a) with the lamp off, $I_{dark}$; and (b) with the lamp on, $I_{teflon}$.

Since measurements are made in interactance, an opaque reference material is unsuitable. Therefore, a semi-transparent Teflon block is used as a diffuse scattering standard. To ensure that no reflection is measured, two blind holes are drilled into the Teflon block to a depth of approximately 0.5 mm. The ferrules dip into the Teflon block when positioned correctly.

Reference spectra are used in the transformation of any other sample intensity, $I_{sample}$, into reflectance using equation:

(1) $$reflectance=\frac{I_{sample}-I_{dark}}{I_{teflon}-I_{dark}}$$

Reflectance can be transformed into absorbance using equation:

(2) $$absorbance=log\left(\frac{1}{reflectance}\right)$$

#### 2.4.3. Metrics

To assess the performance of the models, we apply several quantitative metrics. These are well-known scores and are thus introduced briefly in the following.

For the evaluation of regression models, we establish multiple measures. To start with the coefficient of determination ($R^{2}$), this score indicates the explained share of variance of target variable *y* by the model. We constitute $y_{i}$ as the measured and $\hat{y_{i}}$ is the predicted value. The metric is calculated over the full set of samples *N* and is given by

(3) $$R^{2}\left(y,\hat{y}\right)=1-\frac{\sum_{i=1}^{N}{\left(y_{i}-\hat{y_{i}}\right)}^{2}}{\sum_{i=1}^{N}{\left(y_{i}-\bar{y_{i}}\right)}^{2}},$$

where $\bar{y}=\frac{1}{N}\sum_{i=1}^{N}y_{i}$.

Furthermore, the root mean squared error root mean square error (RMSE) is reported:

(4) $$RMSE\left(y,\hat{y}\right)=\sqrt{\frac{1}{N}\sum_{i=1}^{N}{\left(y_{i}-\hat{y_{i}}\right)}^{2}}.$$

As a final score, the ratio of standard deviation and root mean square error is defined as

(5) $$RPD\left(y,\hat{y}\right)=\frac{\sigma (y,\hat{y})}{RMSE(y,\hat{y})}.$$

In the classification, we evaluate the estimator by computing the accuracy score (ACC), which is defined as

(6) $$Accuracy\left(c,\hat{c}\right)=\frac{1}{N}\sum_{i=1}^{N}1(\hat{c_{i}}=c_{i}),$$

where 1(x) is the indicator function, $\hat{c_{i}}$ and $c_{i}$ are the predicted and corresponding true classes, respectively. The sum of correct predictions is divided by the total number of samples *N*.

### 2.5. Benchmarking

We used a Felix Instruments F750 as a comparison for our OpenVNT instrument. The F750 is established in both science and commercial field applications [9]. Both competing set-ups were validated by sampling white grapes. We chose white grapes because of their availability, small size, and simple juice extraction process. For collecting target variables, we used a digital PCR-DRC2 refractometer manufactured by PCE-Instruments to determine the Brix value of the grape juice. Subsequently, we built chemometric models from both datasets and ground truth measurements. For this purpose, we trained one PLSR model per instrument to predict the Brix values from spectral data.

### 2.6. Samples

White grapes were purchased from the grocery store and used unwashed. In total, 115 grapes were stored and examined at room temperature. All individual grapes were removed from the vine by cutting their stalk. Afterwards, each grape was numbered with a pencil. All grapes were used for recording a time series at different days. Out of 115 grapes, 93 were also analysed destructively. The sample size varied from 20 to 30 grapes per day. Measurement days were day 1, 3, 5, 6, 8 and 10 of storage. Thus, the number of samples decreased during the examination. Subsequently, we recorded spectral measurements with the OpenVNT and the F750 instruments, holding a single grape against the probe head and triggering a measurement. Lastly, we used the refractometer to destructively examine the grapes for the regression model. Approximately three drops of juice from a squeezed berry were extracted to determine the respective Brix value. The measured value was read and assigned to the corresponding spectral data. The day of measurement was assigned to the corresponding spectral data.

### 2.7. Spectrometer

We configured the OpenVNT instrument to average 10 spectra for each measurement from the internal VIS and NIR sensors. The VIS sensor covered a wavelength range from 445 to 1062 nm and the NIR sensor covered a wavelength range from 877 to 1693 nm. A semi-transparent Teflon block (20 × 10 × 10 mm) served as a diffusive spectral reference material, as can be seen in Section 2.4.2. Our comparison instrument, the F750 sensor, covered a wavelength range from 400 to 1064 nm. The F750 firmware automatically selected 5–20 spectra for averaging. In order to keep external influences, e.g., ambient light, temperature fluctuations, etc., on the measurements as small as possible, all measurements were performed under comparable conditions in a room with artificial light. In order to be able to mathematically eliminate the possible influence of ambient light through a sample, dark spectra were also recorded for each measurement and taken into account when calculating the reflectance. We transformed all spectral data from intensities into reflectance for further use, as described in Equation (1).

### 2.8. Modelling

The software versions we used for data processing, model building, and visualisation were: Python in version 3.9.7 with the pandas 1.4.0, numpy 1.21.5, scikit learn 1.0.2, scipy 1.8.0, and matplotlib 3.5.1 packages. We designed our methods for model training based on spectra data and ground truth for the scikit learn pipeline api. The following methods for preprocessing spectral data were implemented:

(1) Application of a fruit-specific spectrum filter based on all spectral data;
(2) Transformation of reflectance into absorbance;
(3) Smoothing of the spectrum using a Savitzky–Golay filter without derivation;
(4) Selection of a wavelength range for modelling;
(5) Normalisation of the spectrum to zero by subtraction at a certain wavelength;
(6) Application of a Savitzky–Golay filter with derivation.
  After preprocessing, we define the estimator for modelling and the model selector:
(7) Compute the sklearn PLSR or the LDA with its hyperparameter;
(8) Test the fitted estimator using a k-fold cross validation.

A brute force approach using grid search cross-validation selects which of the aforementioned methods and hyperparameters are chosen for the final pipeline set-up.

We split the dataset in a ratio of 80:20—80% of the data were used to train models, and the remaining 20% were used for validation.

We consider the best model to be the one whose root mean square error of cross validation (RMSECV) is minimal or the accuracy score of cross validation (ACCV) is closest to one. The corresponding hyperparameters are considered optimal. Based on the collected data, we calculated PLSR and LDA models. We chose the $R^{2}CV$ and ACCV as a measure of the goodness of the instrument.

## 3. Results

The number of outliers we had to remove from the 93 grape datasets depended on the spectrometer. We removed spectral data that were atypical using spectral data filters before modelling. After modelling, possible outliers based on the residuals were not removed and the model was not recalculated. Hereby, the quality of a model based on the measured values of OpenVNT is shown unadorned. This determined the number of samples for modelling. Table 2 shows the number of outliers and samples used for modelling, the mean, median, standard deviation (sd), minimum (min), and maximum (max) Brix value of the respective samples.

**Figure 2:**
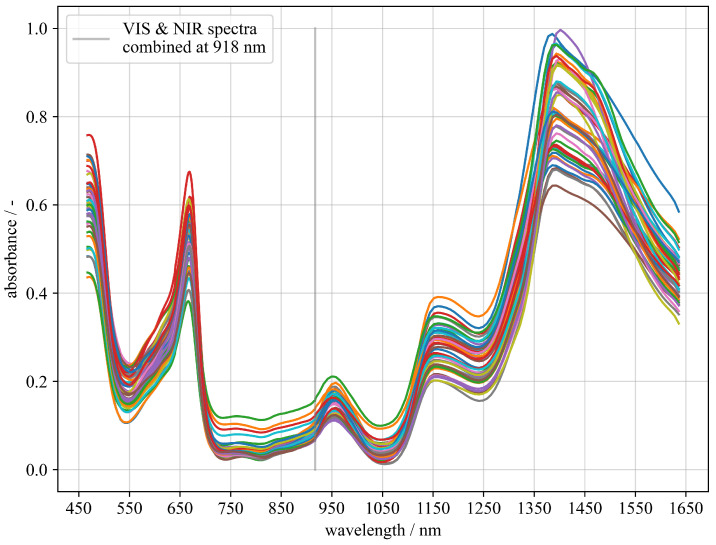
Preprocessed OpenVNT spectra of Vis29 and Nir22 (coloured lines), combined at 918 nm, vertical grey line.

We merged the spectral data from Vis29 and Nir22 at the wavelength 918 nm, after both sets of spectra were independently filtered and smoothed. For this purpose, the difference to the corresponding VIS spectrum at 918 nm was subtracted from each NIR spectrum, so that the spectra merge at this point. Figure 2 shows the combined spectra transformed to absorbance in order to briefly discuss some of the spectral features. The shape of the spectra is typical for white grapes. Absorbance bands at 450 and 660 nm in VIS region are especially characteristic for chlorophylls [38]. Absorbance in the VIS spectrum of electromagnetic radiation is related to molecular electronic transitions. Absorbance in NIR at approximately 760, 960, 1200, and 1400 nm is also typical and assigned to the absorbance of electromagnetic radiation by molecules due to vibrational energy transitions. Typically, water O–H vibrations’ first and third over tone absorb at approximately 760, 969 and 1450 nm [39,40]. Alcohols and carbohydrates with their O–H and C–H bonds also absorb NIR radiation in that region [41].

Table 3 shows the hyperparameters selected by the grid search cross-validation in order to achieve a minimal RMSECV. In the case of the Vis29 spectral data, strong smoothing with a large window length of 19 was the optimal configuration. Even if smoothing parameters for the F750 are unknown, the comparison of spectral data (not shown) also implied strong smoothing. The algorithm selected no smoothing for pure Nir22 data. Our combined spectral data were already smoothed before merging according to the described parameters. No further smoothing by the algorithm was selected. In all cases, reflectance and wavelength normalisation were selected. F750 data also seem to be normalised at approximately 767 nm. Except for the Vis29 and Nir22 data, the algorithm selected smoothing and the first derivative in order to optimise RMSECV.

**Figure 3:**
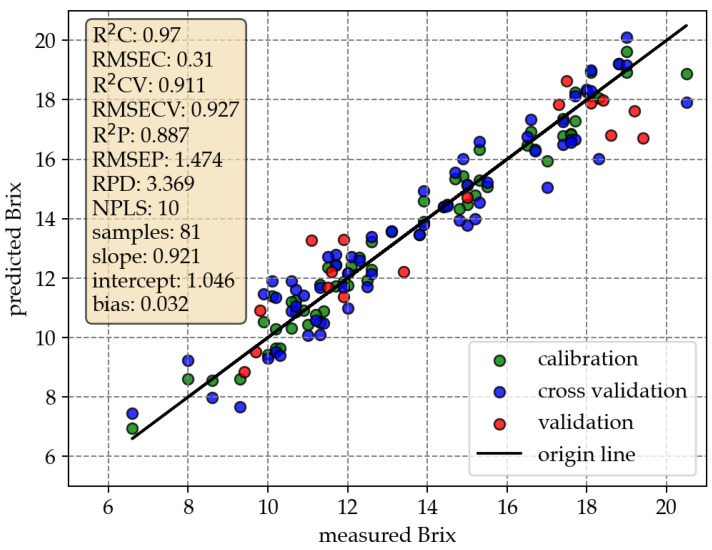
Correlation plot based on OpenVNT Vis29 spectral data including specific results. Where applicable, the unit % Brix is used.

Figure 3 contains the correlation diagram of the estimated versus measured Brix values based on the Vis29 data. Predicted Brix values are closely distributed along the slope 1 line. This indicates, in most cases, the precise prediction of the Brix value from spectral data. Key model parameters are also shown. The algorithm selected 10 number of PLSR components (NPLS). With $R^{2}CV$ of 0.93, the model exhibits high linearity. The RMSECV is 0.93% Brix and the ratio of standard deviation and root mean square error (RPD) of 3.4 implies that the model has acceptable predictive power, since the RPD is greater than 3 [2]. The parameters of the regression slope (0.92), intercept (1.05) and bias (0.03) are given. These values indicate that the model can predict with acceptable accuracy over a wide range of Brix values.

Despite the small number of samples, validation was performed with 20% of all data. Corresponding results can also be seen in Figure 3. The coefficient of determination of validation (*R*^2^*P*) is given as 0.89 and root mean square error of validation (RMSEP) as 1.47% Brix. Accordingly, the prediction accuracy based on data unknown to the model is worse. This is not surprising considering the quantity of data. Nevertheless, the results are evidence of an acceptably functioning OpenVNT.

All other results are summarised in Table 4.

Table 4 summarises our results. From an application perspective, all models perform in a comparable manner. The OpenVNT instrument with its multiple spectrometers achieve results close to the benchmark F750. The Vis29 spectrometer performs better than the Nir22 spectrometer. A physical reason here may be the lower penetration depth of radiation of a longer wavelength. In addition, InGaAs sensors are more sensitive to temperature changes than Si sensors. During operation, the temperature in the case of the OpenVNT increases, which leads to a shift in pixel intensities. In contrast to the F750, where temperature compensation is applied, OpenVNT has no temperature compensation implemented due to simplicity. This might result in a slightly higher NPLS for Nir22. The combined spectral data of Vis29 and Nir22 give the best result for the OpenVNT, but with 18 NPLS the model is complex. In general, the NPLS is a relatively high. This might result from sampling grapes for the regression model on different days, resulting in more variance to explain. Finally, the model based on F750 data has the best result in the experiment.

In a similar way, a model for estimating the storage day was created using OpenVNT only. Instead of PLSR, a LDA was implemented as the estimator. The rest of the pipeline was unchanged. As a metric, ACCV was chosen.

The purpose of this experiment is to demonstrate that it is possible to use OpenVNT to observe time-dependent changes, e.g., the maturity, of samples during a storage period. The data are based on snapshots of the individual samples and therefore do not allow a generally valid description and classification of the physiological age of the samples. The classification classes correspond with their names to the days of measurement, not to the actual age of the samples.

**Figure 4:**
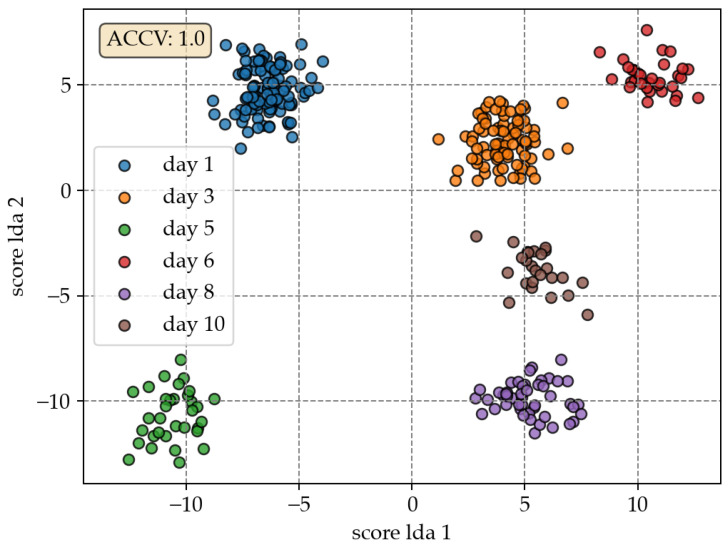
LDA scores based on OpenVNT Vis29 spectral data.

Results of classification by LDA based on spectral data by Vis29 are as follows. Figure 4 shows a score plot of the first two of five number of LDA components (NLDA). As can be seen, samples are well clustered and those are well separated from each other. With an ACCV of 1.0, all spectral samples of OpenVNTs Vis29 are classified correctly. Therefore, hyperparameters reflectance, double smoothing (wavelength 13, poly-order 2) and wavelength normalisation at 546 nm was selected by the grid search cross-validation. The performance of the LDA based on spectral data of Nir22 is ACCV of 0.89. This implies that NIR is less useful than the VIS spectral range. No prepossessing was chosen by the grid search. The combined spectral data of Vis29 and Nir22 results ACCV of 0.99. Combined spectral VIS-NIR data undergo double smoothing (wavelength 5, poly-order 2) and wavelength normalisation at 580 nm.

Nevertheless, the comparison of the PLSR result indicated by $R^{2}CV$ and RMSECV shows that OpenVNT provides spectral data suitable for building PLSR models with high quality. In addition, the ACCV indicates that classification models with high accuracy can also be build.

## 4. Discussion

### 4.1. Instrument

The OpenVNT instrument meets all the requirements we defined in Section 2. Compared to some commercial NIR scanners, the OpenVNT is also a small and light instrument (see Table 5). It can be operated with one hand. Data can be stored on the device and communicated wirelessly into data flows. The ESP32 is powerful enough to apply machine learning models to spectral data on the device if desired. Results could be communicated immediately to the user using the display. Our OpenVNT instrument can be integrated into a downstream process. With a full open design and software stack, our major advantage is that of data sovereignty.

As discussed in Section 3, it is noteworthy that OpenVNT measurements contained more outliers than F750 measurements. One reason for the outliers could be the incorrect operation of the instrument. Removing samples before measurements are completed from the probe head leads to faulty spectral data. In contrast to the F750, OpenVNT requires the sample to be held still during the entire measurement. With the F750, the sample lies still on the probe head. Another difference is that the OpenVNT measures single spots with a diameter of approximately 200 µm for each spectrometer. This makes the OpenVNT more susceptible to sample inhomogeneity. F750 collects diffuse reflectance by lens from an circular area with a radius of approximately 1 cm. The results of OpenVNT might be optimised by changing some of its configuration parameters. For example, SNR can be increased by increasing the number of scans to average. The hardware of OpenVNT could be optimised using a collimator lens. This would help to collect more diffuse reflectance from a sample. Another improvement would be a sample holder that holds a still sample in place. Unfortunately, we were not able to decrease the measure time to 2 s, due to the limitations of the software stack at that time. Thus, both spectrometers can only be controlled consecutively and not simultaneously. If one spectrometer is used, only measurement time can be reduced to 2–3 s.

### 4.2. Software

Since OpenVNT software at present in version 1.0.0 is completely open source, this allows customisation to potential users. Its code is written in MicroPython, which makes it relatively easy to read and change compared to high-level languages and embedded development set-ups. The advantage of the layered structure of the software is that the interaction of functions is easy to understand, adapted or even replaced with different hardware or mock components. If changes are required, then the software does not need to be changed in its entirety. For example, in order to adapt the software to a respective application, small changes can be made via a configuration file or by changing the measurement procedure. Another big advantage is that software also works with one spectrometer only. Thus, the more expansive Nir22 can be left out for using Vis29 only. The hard- and software can easily be extended to include different or additional sensors. For example, a temperature compensation could be implemented to further improve the measurement results.

### 4.3. Validation

Results of the validation in Section 3 show that the OpenVNT delivers spectral data that can be used for regression and classification models. All model parameters are comparable with those of the F750 instrument. Compared to results by other instruments and laboratory setups from the literature, the VNT performs well [42,43]. For example, Goisser et al. [12] reported an $R^{2}CV$ of 0.95, RMSECV of 0.57, NPLS of 5 using F750 or $R^{2}CV$ of 0.94, RMSECV of 0.51 and NPLS of 7 using Scio.

## 5. Conclusions

In this paper, we present OpenVNT: an open platform for capturing, transmitting and analysing spectral measurements. Our proposed OpenVNT instrument combines the spectral measurements of two spectrometers, covering a combined wavelength range from 400 to 1700 nm. We provide open build instructions, schematics, firmware, and data analysis software. In a comparison with the Felix Instruments F750, OpenVNT reached comparable results at a tenth of the cost and full data sovereignty. OpenVNT allows the free configuration of relevant parameters, e.g., the number of spectra averaged per measurement, and leaves users in control of data recipients, but provides an open, customisable data analysis toolbox.

Obtaining information about material properties in a nondestructive and fast fashion is of interest for many application domains. Our focus was on applications in the food supply chain, e.g., assessing the quality (Brix) and remaining shelf-life (days of storage) of fresh fruits. Other industrial IOT applications of spectroscopy include the inspection of incoming goods, e.g., fabric, raw materials, plastics, and drugs. Spectroscopy is also used to sort different materials in recycling, e.g., separating different kinds of plastics, and in healthcare.

Lately, many business models rely on data in addition to the hardware as a secondary income source leading to walled gardens. Closed platforms hamper scientific research and make the seamless integration of technologies costly. By providing raw data and open resources, we hope to contribute to research projects and industrial applications.

## Figures and Tables

**Table 1:** Hardware components and their approximate prices of the OpenVNT instrument.

Device	Name, Specifications and Approximate Costs	Source
VIS spectrometer	UVVis29, Si-Photodetector-Array, EUR 700	Dr. Licht GmbH, Nümbrecht, Germany
NIR spectrometer	Nir22, InGaAs-Photodetector-Array, EUR 2500	Dr. Licht GmbH, Nümbrecht, Germany
Light source	TRU Components 1590265, 3.6 V, 1.22 W, socket Bi-Pin 1.27 mm, EUR 3	Conrad Electronic SE, Hirschau, Germany
Reflector	Model Mentor 2450.5100, Polycarbonate for LED 4.9 mm, EUR 1	Re-In Retail International GmbH, Nuremberg, Germany
Microcontroller	M5Stack FIRE, ESP32 with PSRAM 2.0, EUR 70	Eckstein GmbH, Clausthal-Zellerfeld, Germany
Battery	18500 Li-Ion Battery, 3.7 V, 2300 mAh, incl. PCB P1850C3, EUR 20	Akkuteile, Aschaffenburg, Germany
Boost converter	Adafruit PowerBoost 1000 Charger, Lipo USB Boost, EUR 30	Reichelt Elektronik GmbH & Co. KG, Sande, Germany
USB-C	Adafruit USB-C Breakout Board, Downstream Connection, EUR 4	Eckstein GmbH, Clausthal-Zellerfeld, Germany
Switch	Adafruit Breadboard SPDT Slide Switch, EUR 2	Eckstein GmbH, Clausthal-Zellerfeld, Germany

**Table 2:** Number of outliers and sample statistics. Where applicable, unit % Brix.

Spectrometer	Outlier (s)	Samples	Mean	Median	Sd	Min	Max
Vis29	12	81	13.8	13.3	3.3	6.6	20.5
Nir22	0	93	14.3	14.4	3.5	8.0	25.1
Vis29 and Nir22	12	81	13.8	13.1	3.3	6.6	19.4
F750	0	93	14.4	14.5	3.7	6.6	25.1

**Table 3:** Overview of algorithm-selected hyperparameters.

Spectrometer	Savitzky–Golay Filter Smoothing		Savitzky–Golay Filter Smoothing and Derivative			Wavelength Normalisation
	Window Length	Poly-Order	Window Length	Poly-Order	Derivative	
Vis29	19	2	11	2	1	968 nm
Nir22	-	-	13	2	1	1200 nm
Vis29 and Nir22	-	-	3	2	0	1128 nm
F750	Unknown	Unknown	19	3	1	Unknown

**Table 4:** Comparison of models. First three lines: our hardware set up, last line: F750 by Felix instruments. Where applicable, the unit % Brix is used.

Spectrometer	*NPLS*	$R^{2}$ *C*	*RMSEC*	$R^{2}$ *CV*	*RMSECV*	$R^{2}$ *P*	*RMSEP*	*RPD*
Vis29	10	0.97	0.31	0.91	0.93	0.89	1.47	3.37
Nir22	13	0.90	1.25	0.80	2.53	0.91	1.49	2.27
Vis29 and Nir22	18	0.97	0.33	0.86	1.42	0.92	1.0	2.72
F750	12	0.97	0.35	0.90	1.34	0.96	0.73	3.11

**Table 5:** Comparison of instruments by wavelength range and form factor.

Instrument	Wavelength Range (nm)	Size (cm)	Weight (g)	Application
F750	350–1100	18 × 12 × 5	1050	fruit quality
H100	650–950	11 × 16 × 17	450	fruit quality
Scio	740–1040	7 × 4 × 3	35	food quality
OpenVNT	350–1700	15 × 6 × 4	300	universal

## Data Availability

Publicly available datasets were analyzed in this study. This data can be found here: https://mygit.th-deg.de/vnt.
